# Prevalence and molecular characterization of *Salmonella* isolated from wild birds in fresh produce environments

**DOI:** 10.3389/fmicb.2023.1272916

**Published:** 2023-11-07

**Authors:** Jared C. Smith, Sofia Varriano, Kerrie Roach, Zach Snipes, Joshua L. Dawson, Justin Shealy, Laurel L. Dunn, William E. Snyder, Nikki W. Shariat

**Affiliations:** ^1^Departments of Population Health and Microbiology, University of Georgia, Athens, GA, United States; ^2^Department of Entomology, University of Georgia, Athens, GA, United States; ^3^Department of Plant Industry, Clemson University Extension, Charleston, SC, United States; ^4^Fort Valley State University Extension, Fort Valley, GA, United States; ^5^College of Agricultural and Environmental Sciences, University of Georgia Extension, Athens, GA, United States; ^6^Department of Food Science and Technology, University of Georgia, Athens, GA, United States; ^7^Center for Food Safety, University of Georgia, Griffin, GA, United States

**Keywords:** *Salmonella*, produce, wild birds, food safety, WGS

## Abstract

Wild birds pose a difficult food safety risk to manage because they can avoid traditional wildlife mitigation strategies, such as fences. Birds often use agricultural fields and structures as foraging and nesting areas, which can lead to defecation on crops and subsequent transfer of foodborne pathogens. To assess the food safety risk associated with these events, wild bird feces were collected from produce fields across the southeastern United States during the 2021 and 2022 growing seasons. In total 773 fecal samples were collected from 45 farms across Florida, Georgia, South Carolina, and Tennessee, and 2.1% (*n* = 16) of samples were *Salmonella-*positive. Importantly, 75% of *Salmonella* were isolated from moist feces, showing reduced *Salmonella* viability when feces dry out. 16S microbiome analysis showed that presence of culturable *Salmonella* in moist feces correlated to a higher proportion of the Enterobacteriaceae family. From the *Salmonella*-positive samples, 62.5% (10/16) contained multi-serovar *Salmonella* populations. Overall, 13 serovars were detected, including six most commonly attributed to human illness (Enteriditis, Newport, Typhimurium, Infantis, Saintpaul, and Muenchen). PCR screening identified an additional 59 *Salmonella*-positive fecal samples, which were distributed across moist (*n* = 44) and dried feces (*n* = 15). On-farm point counts and molecular identification from fecal samples identified 57 bird species, including for 10 *Salmonella*-positive fecal samples. Overall, there was a low prevalence of *Salmonella* in fecal samples, especially in dried feces, and we found no evidence of *Salmonella* transmission to proximal foliage or produce. Fecal samples collected in farms close together shared highly related isolates by whole genome sequencing and also had highly similar *Salmonella* populations with comparable relative frequencies of the same serovars, suggesting the birds acquired *Salmonella* from a common source.

## 1. Introduction

*Salmonella enterica* is a leading contributor of bacterial foodborne illness in the United States (Scallan et al., 2011; Tack et al., 2019). While *Salmonella* is an enteric pathogen, it can be found in non-host environments, such as surface water and soil, as well as on produce (Critzer and Doyle, 2010; Gorski et al., 2011; Strawn et al., 2013; Reddy et al., 2016; Bardsley et al., 2021; Deaven et al., 2021), where it can survive and cause outbreaks (CDC, 2023). Consumption of contaminated produce causes an estimated 44.2% of salmonellosis cases in the United States [The Interagency Food Safety Analytics Collaboration (IFSAC), 2022]. In produce, contamination can occur through water, soil, equipment, personnel, and wildlife introduction events (Alegbeleye et al., 2018; Rodrigues et al., 2019; Devarajan et al., 2021, 2023). Because produce is often eaten raw and post-harvest kill steps are limited, there is a significant need to understand and mitigate potential sources of contamination. The Standards for the Growing, Harvesting, Packing, and Holding of Produce for Human Consumption (Produce Safety Rule, 74354, 2015) went into effect in 2016 as a part of the Food Safety Modernization Act. This rule set the first federally mandated standards for the safe production of fruits, vegetables, and nuts, and includes requirements for microbial quality of production and postharvest water, soil amendments, cleaning and sanitation practices, worker training and hygiene, and wildlife mitigation in order to reduce the likelihood of foodborne pathogen-contamination to produce. While many of these standards have clear guidelines, wildlife mitigation is often limited to physical barriers to prevent foraging from deer, raccoons, and other land animals (Hamilton et al., 2015). These precautions do little to prevent the intrusion of birds, which can easily fly into fields to forage for plants, insects, or small rodents. Birds are a further challenge as they can become accustomed to deterrents and often fly long distances while migrating (Rivadeneira et al., 2018; Elsohaby et al., 2021).

Wild birds are known to carry foodborne pathogens, including *Salmonella enterica* subspecies *enterica* (Tizard, 2004). Studies performed in the western and southwestern United States found *Salmonella* prevalence in wild birds at 0.5–6.5% (Gorski et al., 2011; Rivadeneira et al., 2016; Navarro-Gonzalez et al., 2020). Additionally, flocks of wild bird can spread disease among individuals when congregating at common food and water sources (Hernandez et al., 2016). Outside of explosive mortality events caused by *Salmonella* serovar Typhimurium (Hernandez et al., 2012), *Salmonella* does not typically elicit symptoms in wild birds, so healthy carriers can transmit this pathogen without suffering from salmonellosis (Prosser et al., 2011). Transmission of pathogens from birds to produce can occur through defecation when birds are flying over fields or foraging for food. An outbreak of *Salmonella* serovar Typhimurium in 2009 found matching strains in birds, peanut crops, and human clinical cases (Hernandez et al., 2012). While birds can benefit farms by providing services like natural pest control (Karp et al., 2013), their habituation in production environments could play a role in the transmission of foodborne pathogens via fresh produce.

*Salmonella enterica* is a diverse species, consisting of over 2,600 distinct serovars that are categorized by their unique O (somatic) and H (flagellar) antigens (Grimont and Weill, 2007; Issenhuth-Jeanjean et al., 2014). Genomic diversity between these serovars has led to differences in host specificity, pathogenesis, and antibiotic resistance profiles (Uzzau et al., 2000; Cheng et al., 2019). While some serovars are most typically found in a small number of reservoirs (e.g., serovar Enteritidis is most closely linked to poultry), others, such as serovar Typhimurium, are ubiquitous and found in a variety of different hosts. Further, *Salmonella* is often detected in food animal production systems and the environment as mixed populations of multiple serovars (Deaven et al., 2021; Siceloff et al., 2021, 2022; Obe et al., 2023). In some instances, low frequency serovars in these populations may have greater potential impacts on public health when they have clinically relevant antimicrobial resistance profiles (Siceloff et al., 2022) or are more often associated with human illness (Deaven et al., 2021). Traditional isolation techniques that rely on picking a small number of colonies from selective agar are unable to resolve complex multi-serovar *Salmonella* populations (Cason et al., 2011). This hurdle is overcome by deep serotyping approaches such as CRISPR-SeroSeq, which can resolve the relative frequencies of multiple serovars in a single sample (Thompson et al., 2018).

In this study, we investigated the role of wild birds in the transmission of *Salmonella* to produce foliage in the southeastern United States. This study region includes more than 12 million acres of cropland (CroplandCROS, 2022) where produce such as tomatoes, peppers, eggplant, and other fruit, vegetable, and nut crops are significant economic contributors. Wild bird feces were collected from produce fields over a two-year period and cultured for *Salmonella*. Deep serotyping and whole genome sequencing were performed to assess *Salmonella* populations and to estimate source attribution. Additionally, wild bird species were identified with both physical and molecular techniques to associate pathogen transmission risk.

## 2. Materials and methods

### 2.1. Site selection and overview of study design

To study the impact of wild bird activity upon produce contamination, 45 different farms across the southeastern United States (Tennessee, Georgia, Florida, and South Carolina) were visited between 1–6 times (average 2.4 visits/farm). Produce grown on these farms included peppers (bell, banana, and jalapeño), eggplant, cucumbers, tomatoes, squash, grapes, pole beans, and okra. These above ground produce were chosen because they pose a greater risk for human illness should they be contaminated, as many are often eaten raw. Additionally, selecting produce growing above ground reduced the incidence of identifying contamination from on-ground sources, such as soil, or rodents or other small wildlife that primarily forage on the soil surface. Farms in this study were diverse and included organic and conventional farms, commercial and family-run operations, mono- and polyculture farms, and some had livestock on and around the farm. To best measure the effect of seasonality on the prevalence of *Salmonella*, repeated sample collections were completed at farms, up to three times per sampling season (May–October), where possible. During each sampling visit, crops around the perimeter and the interior of the fields were inspected to identify wild bird fecal samples. When fecal samples were identified, the leaf containing the feces was removed and homogenized for culturing *Salmonella*. To evaluate the necessity of exclusion zones encouraged by groups such as the Leafy Green Marketing Agreement (LGMA), surface swabs of a piece of produce beneath the fecal sample and from the leaf of a neighboring plant downwind were also collected. *Salmonella* was first identified by culture. Samples found positive for *Salmonella* culture were then further analyzed with additional molecular tools (e.g., PCR, WGS, and CRISPR-SeroSeq). The culture-negative samples were then analyzed by a *Salmonella* PCR.

### 2.2. Sample collection

Fecal samples were collected between sunrise and 11 am to capture on-field bird activity while also limiting UV exposure and reducing the opportunity for desiccation. Upon arriving at a farm, sampling was conducted around the perimeter of each field, followed by a step-wise sampling through the interior of the field. On smaller farms (or small (<1 acre) fields on a large farm), all individual rows were surveyed. When a fecal sample was identified, it was visually scored for moisture as either 1 (moist) or 0 (dry) as an indicator of freshness. Then, the leaf containing the fecal sample was removed, inserted into 2 mL buffered peptone water (BPW, Hardy Diagnostics, Ohio, USA) recovery media, and placed on ice until culturing (within 24 h). Because the fecal samples were small and because in some cases removing them from the leaf would lose some of the fecal material, the entire leaf was removed from the plant and then the portion containing just the feces and the leaf material directly under the feces were isolated and collected. To test for transmission of *Salmonella* from the fecal sample, the surface of a piece of produce under the leaf was swabbed, along with a leaf of a neighboring plant downwind from the fecal sample. These swabs were collected by soaking a sterile cotton ball in 3 mL of BPW and using sterile forceps to drag it across the top and bottom of the neighboring leaf and across the entire surface of the produce. Swabs were placed in a cooler with ice packs and stored at 4°C for no more than 24 h or until culturing could begin in the laboratory.

### 2.3. *Salmonella* culturing

Fecal samples were homogenized by hand into the 2 mL of recovery media. For *Salmonella* isolation, 750uL of the homogenate was transferred into a culture tube containing 9.25 mL BPW and incubated at 42°C for 24 h. Then, this was sub-inoculated into 9 mL Tetrathionate (TT, Neogen Diagnostics, Michigan, USA) and 9.9 mL Rappaport-Vassiliadis (RV, Hardy Diagnostics, Ohio, USA) selective enrichment broths in parallel and incubated for 24 h at 37°C before being streaked onto Xylose Lysine Tergitol-4 agar plates (XLT-4, Hardy Diagnostics, Ohio, USA). The plates were incubated at 37°C for 24 h and inspected for black colonies as an indicator of presumptive *Salmonella* colonies. If no H_2_S-positive colonies were present, the plates were re-incubated for another 24 h. Up to 2 colonies from each sample were selected and were re-streaked onto XLT-4 for isolation if needed. *Salmonella* isolates were grown in Luria Broth (LB, Hardy Diagnostics, Ohio, USA) where aliquots were used to make frozen glycerol stocks and for DNA isolation. If we observed presumptive *Salmonella* colonies, we then returned to the swabs from produce and neighboring foliage and cultured these using the same protocol.

### 2.4. DNA isolation and *Salmonella* PCR screen

The total genomic DNA was isolated from 500uL of the fecal/recovery media homogenate using the Genome Wizard kit (Promega, Wisconsin, USA), with the additional step of grinding the fecal pellet with a sterile mortar and pestle to disrupt the fecal particles before beginning the extraction. Prior to any PCR (i.e., for *Salmonella* or for COI), DNA from this fecal/recovery media homogenate was screened with an internal amplification control (IAC) PCR to identify the presence PCR inhibitors (Rosenstraus et al., 1998). The primers for IAC PCR were IAC_F (5’-AGTTGCAGTGTAACCGTCATGT-3′) and IAC_R (5′- TCGACGAGACTCTGCTGTTAAG-3′) and the IAC template control sequence was IAC (5’-AGTTGCAGTGTAACCGTCATGTACCAGTAATCTGCGTCGCACGTGTGCACCTAGTCTA ATCACTTATGACTCAGATAACTTAACAGCAGAGTCTCGTCGA-3′). For each reaction, the following components were mixed: 39.5uL sterile water, 5uL 10x Taq Buffer, 0.5uL 10uM forward primer, 0.5uL 10uM reverse primer, 0.3uL 100 mM dNTPs, and 1 U Taq polymerase, before 2uL of bird fecal DNA was added as template. Cycle conditions were as follows: 95°C for three minutes followed by 40 cycles of 95°C for 30 s, 56°C for 30 s, and 72°C for 30 s. This was followed by a final elongation of 72°C for two minutes and resting at 4°C. PCR products were visualized by gel electrophoresis. Where there was no amplification, suggesting the presence of PCR inhibitors, a 1:10 dilution of the bird fecal sample DNA was made, and the PCR repeated. In this study, nearly 10% (*n* = 75) of samples contained PCR inhibitors as shown by the IAC PCR. This inhibition was resolved when the template was diluted 10-fold in molecular grade water, and this dilution was used for all subsequent PCRs.

For the *Salmonella* screening, an *invA* PCR was used (Rahn et al., 1992). In this PCR, primers – InvA_F1 (5’-AACGTGTTTCCGTGCGTAAT-3′) and InvA_R1 (5′- TCCATCAAATTAGCGGAGGC-3′) were mixed with 38.5uL sterile water, 5uL 10x Taq Buffer, 2uL of 6.25ug/mL BSA, 1uL 10uM forward primer, 1uL 10uM reverse primer, 0.25uL 100 mM dNTPs, and 1 U Taq polymerase before 2uL of bird fecal sample DNA was added as template. Cycling conditions began with an initial melting temperature of 95°C for three minutes followed by 40 cycles of the following: 95°C for 30 s, 56°C for 30 s, and 72°C for 30 s. A final elongation temperature of 72°C for two minutes was completed before resting at 4°C.

### 2.5. *Salmonella* weather analysis

For each site, weather data from the day prior to collection, including total precipitation, average wind, average humidity, and high temperature values were determined using the closest USGS weather stations. To identify relationships between weather variables and moist feces, we conducted a series of binomial generalized linear mixed models (GLMM) using the glmmTMB package V1.1.7 (Brooks et al., 2023) within R V4.1.1. All continuous variables were standardized prior to analysis. Visits nested within farm and year were used as random effects. We ran models using individual variables as a fixed effect in each model and considered different additive configurations of other weather variables. We assessed multicollinearity using the performance package V0.10.3 (Lüdecke et al., 2021) and homogeneity of variance using the DHARMa package V0.4.6 (Hartig and Lohse, 2022); models meeting these assumptions (i.e., VIF < 5 and equally distributed residuals, respectively) were retained for comparison. Models were compared using the Akaike Information Criterion adjusted for small sample sizes (AIC_c_) using the R package AICcmodavg V2.3–2. We considered “top models” as those with ΔAIC_c_ ≤ 2 (Burnham and Anderson, 2002). The same weather stations were used to calculate the monthly average weather values (Supplementary Figure S1).

### 2.6. Whole genome sequencing

Total genomic DNA from *Salmonella* isolates was extracted using a Promega Genome Wizard DNA extraction kit (Promega, Wisconsin, USA) and sequenced on an Illumina MiSeq 500 cycle v2 chemistry kit (Illumina, California, USA). The sequence reads were assembled using SPAdes de-novo assembly (Version 3.15.5) (Bankevich et al., 2012) and the serovar determined using SeqSero 2.0 (Zhang et al., 2019). Sequences were uploaded to Enterobase (Zhou et al., 2020) where sequence types (ST) could be predicted and used to identify related isolates. Phylogenetic relatedness was visualized through GrapeTree and allelic differences were used to identify the closest related source type. The assembled genomes were uploaded to NCBI (Accession numbers SAMN33186945, SAMN33186956, SAMN33186963, SAMN33186964, SAMN33186971, SAMN33186984, SAMN33187804, SAMN33187835, SAMN33187836, SAMN33187842, SAMN33187843, SAMN33187878, SAMN33187961, SAMN33187962, SAMN33187972, SAMN33225914, SAMN37196586, SAMN37196587, and SAMN37196588).

### 2.7. *Salmonella* population analysis

To identify the populations of *Salmonella* within wild bird feces, TT and RV enrichments from *Salmonella* culture positive samples were processed individually by centrifuging 1 mL of each selective enrichment at 14,000 rpm for three minutes. Total genomic DNA was isolated from the resulting pellet using a Promega Genome Wizard Kit and resuspended in 200uL of molecular-grade water. A total of 2 μL of this template was used in the PCR for CRISPR-SeroSeq with primers targeting the conserved direct repeat sequences within *Salmonella* CRISPR arrays (Thompson et al., 2018; Siceloff et al., 2022). Primers also included index sequences which facilitated multiplexed, high throughput sequencing. PCR products were purified using the Ampure system (Beckman Coulter, Indianapolis, IN) and pooled in approximate equimolar ratios. Pooled libraries were sequenced using the Illumina NextSeq 550 platform (Illumina, California, USA) mid output 150 cycle v2.5 kit with single-end reads. A water negative-control and a positive control containing *Salmonella* serovar Enteritidis genomic DNA with a known CRISPR profile were included in the library. Sequence reads were scanned and matched in a local BLAST search to a lab-curated database of over 150 serovars (Siceloff et al., 2022).

Serovars were called only if they contained multiple CRISPR spacers that were unique to that serovar. Where there were sufficient *Salmonella* sequence reads (>1,000 reads) for both the TT and RV enrichments the relative frequency of each serovar was normalized across both enrichments to provide a single serovar profile.

### 2.8. Microbiome analysis

All 16S rRNA Illumina-tag PCR reactions were performed on DNA extracts per the Earth Microbiome Project protocol (Walters et al., 2016). Negative controls (molecular grade water) were processed in parallel with the samples for PCR amplification. PCR products were pooled in batches of ~200 samples each and gel purified on a 2% agarose gel using the QIAquick Gel Purification Kit (Qiagen, Frederick, Maryland, USA). Before sequencing, purified pools were quality checked using an Agilent 2100 BioAnalyzer and Agilent DNA High Sensitivity DNA kit (Agilent Technologies, Santa Clara, California, USA). The purified pools were stored at −20°C, then sequenced using an Illumina MiSeq 500 cycle v2 chemistry kit (Illumina, California, USA). Raw data were processed, analyzed, and quality checked with QIIME2 (Bolyen et al., 2019) before forward and reverse reads were merged and chimeras removed with DADA2 (Callahan et al., 2016). DADA2 was also used to assign sequences to amplicon sequence variants (ASVs) using a pre-trained Silva 132 Database (Quast et al., 2012). MAFFT (Katoh and Standley, 2013) and FastTree (Price et al., 2010) were used to create a rooted phylogenetic tree using representative ASVs. Additionally, a biomarker analysis was completed to identify taxonomic groups that were differentially abundant within groupings of samples (*Salmonella* Culture, *Salmonella* PCR, and No *Salmonella*) using LEfSe (Segata et al., 2011) by normalizing the ASVs with the counts per million method and a differential abundance value of *p* of <0.05 and a log(LDA) score of at least 1.0.

### 2.9. Bird species identification

Wild birds were identified in two ways: physical identification (i.e., point counts) of birds present around and in fields, and molecular identification from feces. Point counts were conducted at all field locations on sample days between 6 and 10 am. One point count was done for every 10 hectares (ha) of sampled field when field conditions and harvesting schedules allowed. Points on the same farm were at least 200 m apart. Points were positioned approximately 90 m away from the edge of fields to overlap with bacterial sampling areas while still capturing birds moving in and out of produce. All birds seen and heard within a 100-m radius during a 10-min period were recorded, along with distance, detection method, and habitat. During the 10 min, birds were counted in sub-periods of three, three, and four minutes. Only new species were counted after the first sub-period to avoid counting the same individual multiple times. Birds flying overhead were excluded unless they were a species that forages aerially (e.g., swallows), in which case a note was made that they were “aerial foraging.” The same observer conducted all counts for both years of sampling. Birds were categorized as in-field if they were observed interacting with produce (e.g., in tunnels, perching on produce stakes, or on produce plants) and other birds were categorized as off-field.

Molecular identification of wild bird species from fecal samples was completed using 2uL of DNA isolated from fecal samples as part of a PCR to amplify the Cytochrome C Oxidase Subunit I (COI). The sequence variability of the COI gene between bird species enables species identification. Many COI PCR assays were attempted, following published protocols (Hebert et al., 2003; Ivanova et al., 2007; Kerr et al., 2007; Joo and Park, 2012), but either did not yield amplicons or failed to produce quality sequences. This PCR used the following primers: COI_F1 (5’-CGCYTWAACAYTCYGCCATCTTACC-3′) and COI_R1 (5′- ATTCCTATGTAGCCGAATGGTTCTTT-3′) (Patel et al., 2010). For each reaction, the following were mixed into a 50uL reaction: 38.5uL sterile water, 5uL 10x Taq Buffer, 2uL 25 mM MgCl2, 1uL 10uM forward primer, 1uL 10uM reverse primer, 0.3uL 100 mM dNTPs, and 1 U Taq polymerase along with adding 2uL of DNA template. The mix was run on the following PCR program: Initial melting of 95°C for four minutes was followed by five cycles of 95°C for 30 s, 59°C for 30 s, and 68°C for 45 s. This was followed by 40 cycles of 95°C for 30 s, 56°C for 30 s, and 68°C for 45 s and a final two-minute elongation step. Appropriately sized amplicons were sequenced in the forward and reverse direction by Eton Bioscience Inc. (Research Triangle Park, NC). SeqMan (Lasergene, DNA Star) was used to assemble the forward and reverse reads into a single sequence, which was then compared to two databases: NCBI BLAST, and the Barcode of Life Database (Meiklejohn et al., 2019) with a 97% nucleotide identity threshold.

## 3. Results

**Figure 1:**
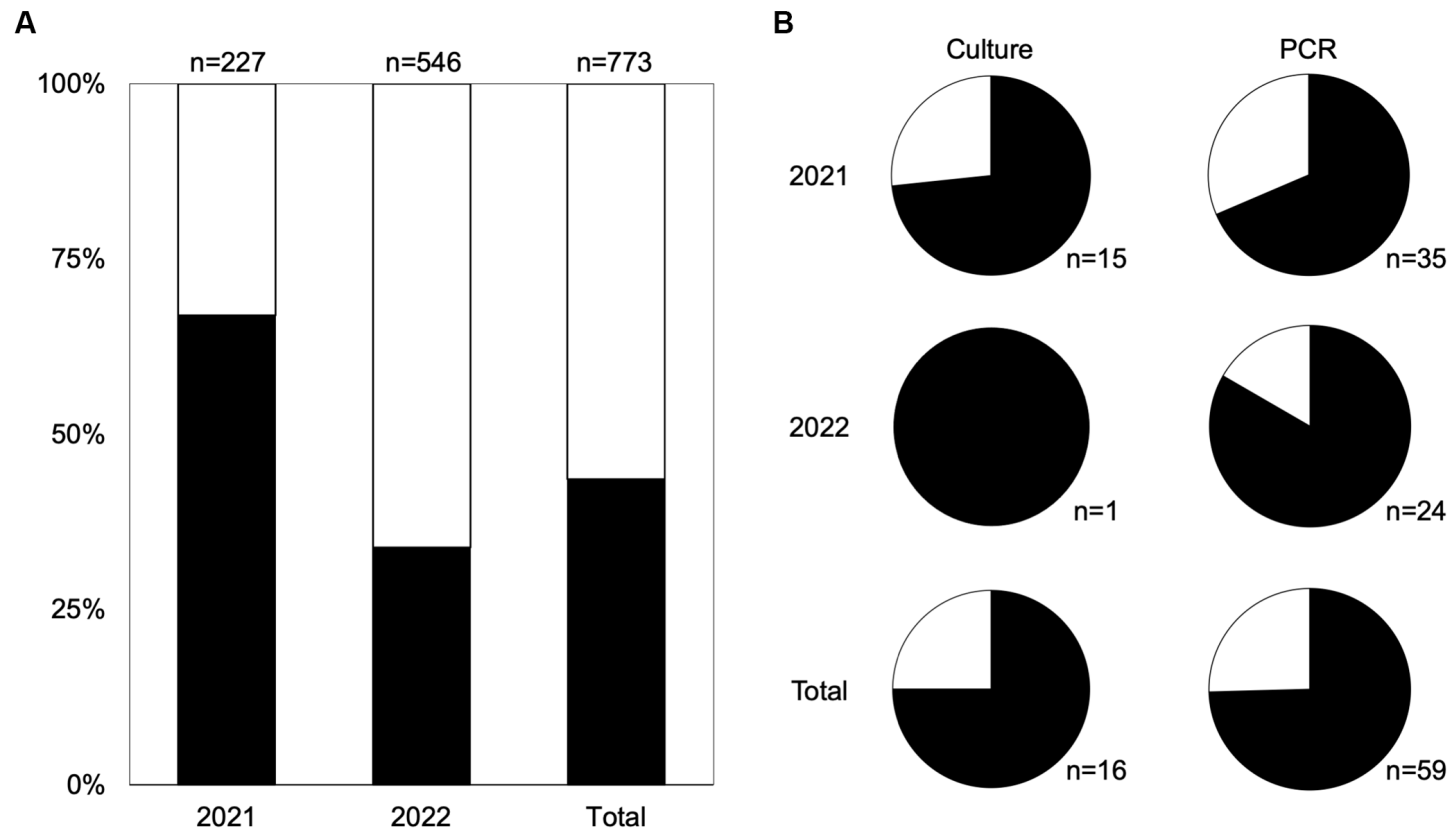
Moist feces support survival of *Salmonella* better than dry feces. **(A)** The distribution of moist (black) and dry (white) feces per year and in total. **(B)** Proportion of *Salmonella-*positive samples in both culture positive (left) and PCR-positive samples (right) and the number of positive samples is indicated below each pie chart. Moist feces are shown in black and dry feces shown in white.

During 2021 and 2022, 109 farm visits were performed across the southeastern United States, including Tennessee (*n* = 4 farms), North Georgia (*n* = 8), South Georgia (*n* = 20), South Carolina (*n* = 10), and North Florida (*n* = 3; Table 1). Farms ranged in size from 1.6–233 acres and included 13 small or independently owned farms (1.6–33.3 acres), as well as 32 large commercial farms (6.95–233 acres). Over the two seasons, 773 fecal samples were collected: 227 samples in 2021 and 546 in 2022. In total, 43.6% (337/773) of fecal samples were scored as moist, including 152 in 2021 and 185 in 2022, while 56.4% (436/773) were scored as dry, including 75 in 2021 and 361 in 2022 (Figure 1A).

**Table 1:** Sampling distribution across the Southeast.

State	Number of farms (Number of visits)	Number of fecal samples collected
TN	4 (16)	218
SC	10 (29)	225
GA-N	8 (31)	235
GA-S	21 (27)	76
FL	3 (6)	19
Total	45 (109)	773

By culture, *Salmonella* was isolated in 16 samples (16/773 total samples; 2.1%); 15 were identified in the first year of collection (15/227, 6.6%) and one was identified in the second year (1/546, 0.2%). Three quarters (12/16) of *Salmonella* samples were recovered from moist fecal samples (Figure 1B). *Salmonella*-positive samples were found in South Georgia (*n* = 10), Florida (*n* = 4), and North Georgia (*n* = 2). There was no recoverable incidence of transmission from fecal samples to produce below leaves with feces, nor to neighboring plants downwind. We screened all samples not confirmed positive by culture using a PCR targeting the *Salmonella invA* gene and detected *Salmonella* in 59 additional fecal samples, bringing the total *Salmonella*-positive samples to 75 (9.7%) (Table 2). Similar to culture-positive fecal samples, *Salmonella* was more commonly detected in the first year of collection, with 16.5% (35/212) of culture-negative samples from 2021 being PCR-positive, while 4.4% (24/545) of culture-negative samples from 2022 were PCR-positive. The proportion of PCR positive samples in moist and dry feces matched the culture data, with three quarters (74.6%, 44/59) of the PCR-positive fecal samples being moist, compared to a quarter from dry feces (25.4%, 15/59) (Figure 1B). Overall, *Salmonella* was significantly more likely to be detected in moist samples than dried samples [χ^2^(1, *n* = 773) = 6.55, *p* < 0.05].

**Table 2:** *Salmonella* prevalence increases with inclusion of molecular detection.

	Fecal Samples	Viable *Salmonella*	Prevalence (%)	Additional PCR Positive	Prevalence (%)	Total	Prevalence (%)
Year 1	227	15	6.6	35	15.4	50	22
Year 2	546	1	0.2	24	4.4	25	4.6
Total	773	16	2.1	59	7.6	75	9.7

Given the positive association between *Salmonella* presence (by culture and by PCR) and moist feces, we used a binomial generalized linear mixed model (GLMM) to explore weather factors that could influence fecal moisture. We identified four top models (i.e., ΔAICc <2) (Supplementary Table S1). Precipitation the day before sample collection was included in three of the top models and was positively associated with moist feces. During most sampling months in 2022, monthly cumulative precipitation was lower than in 2021 (Supplementary Figure S1), which may explain the reduced *Salmonella* detection in 2022. Humidity was also included in three models, and had negative correlations with moist feces. Although temperature did not appear in our models, we expect that high temperatures would contribute to drying the feces. During May–July, the average temperatures were hotter in 2022 than in 2021 in all sampled regions, which, in combination with reduced precipitation may also contribute to the reduced *Salmonella* detection in feces in the second year of sampling.

**Figure 2:**
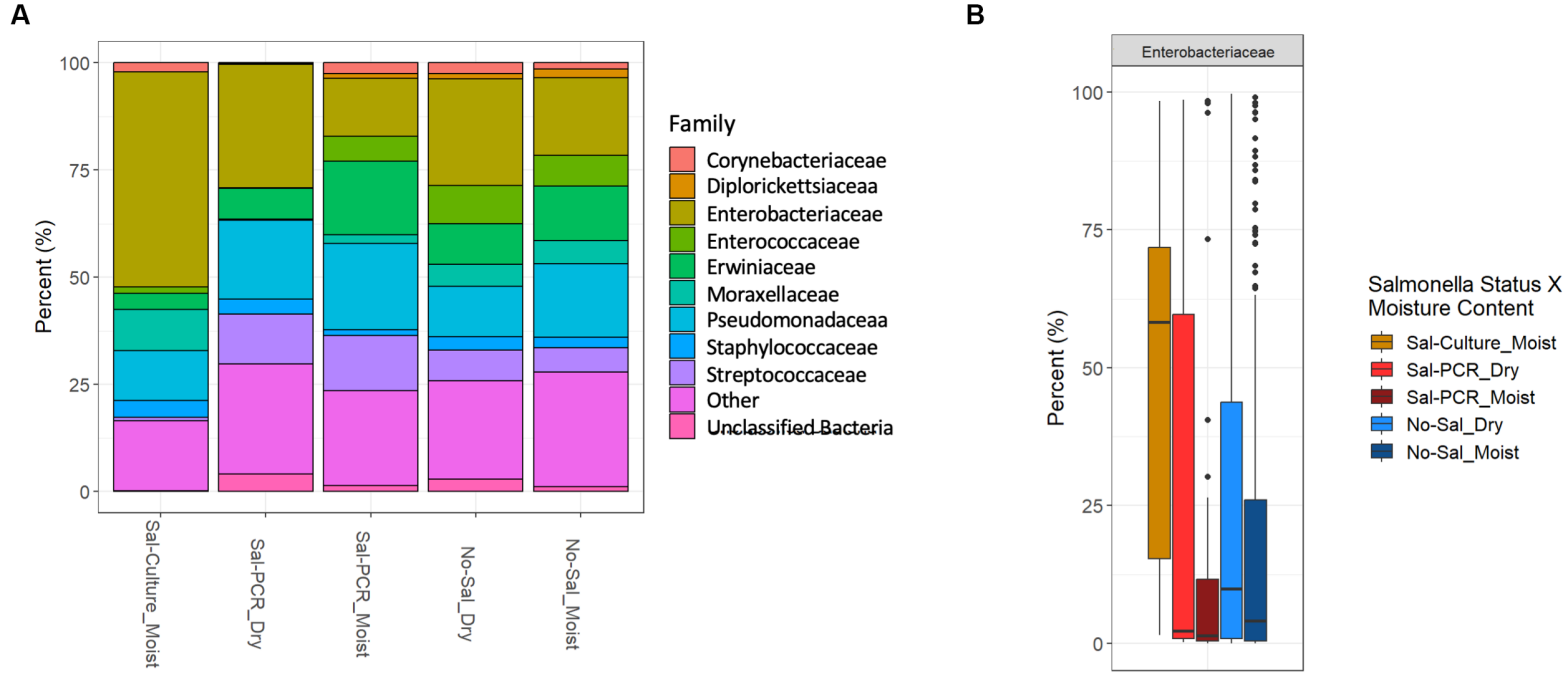
16S sequencing of bird feces shows microbial community differences in *Salmonella* culture positive samples. **(A)** 100% bar graph of mean abundances of the 10 most prominent families identified across the entire dataset are displayed when summarized by *Salmonella* group [*Salmonella* culture positive from moist feces (Sal-Culture_Moist), *Salmonella* PCR positive from dry feces (Sal-PCR_Dry), *Salmonella* PCR positive from moist feces (Sal-PCR_Moist), no *Salmonella* from dry feces (No-Sal_Dry), and no *Salmonella* from moist feces (No-Sal_Moist)]. All taxa outside the top 10 taxa are classified as “Other.” **(B)** Differential relative abundance boxplots of prominent Enterobacteriaceae are displayed with significantly (Wilcoxon Rank Sum test, adjusted value of *p* < 0.05) different pairwise relationships displayed.

We assessed total microbial diversity in each fecal sample (*n* = 773) by 16S rRNA sequencing, with 720 samples passing quality control. Weather variables (precipitation, temperature, humidity, and wind) did not have a strong positive or negative (±0.30) impact to alpha diversity (data not shown). *Salmonella* was not found to affect species richness when comparing culture-only positives or PCR positives to the *Salmonella* negative group (data not shown). Because the number of *Salmonella-*culture positive samples was low, we presented the microbiome data stratified into six different groups, based on *Salmonella* status and fecal moisture. The group containing *Salmonella* culture-positives from dry feces was removed from the groups, as the low number of samples (*n* = 4) reduced significant findings. Within moist feces, the Enterobacteriaceae family was significantly enriched in samples containing culturable *Salmonella* compared to samples containing only molecularly detectable *Salmonella* or no detectable *Salmonella* (Figures 2A,B). This included a significant increase in the *Escherichia-Shigella* genera (these cannot be separated using 16S) in the *Salmonella*-culture group, rather than *Salmonella* (data not shown) (Wilcoxon rank sum test adjusted *p* value <0.05).

Whole genome sequencing was completed on 19 isolates (JSBird1-JSBird19) (Supplementary Table S2), and eight serovars were subsequently identified: Hadar (5 isolates), Give (4), Newport (4), Saintpaul (2), Kentucky (1), Mississippi (1), Muenchen (1), and Typhimurium (1) (Table 3). Using Enterobase, we next searched for related isolates. Four serovar Hadar isolates were closely related to each other (JSBird3-JSBird5 and JSBird10) and to isolates collected from ground turkey meat (within the same HierC2 cgMLST cluster) (Supplementary Figures S2A,B). The fifth serovar Hadar isolate (JSBird11) was more closely related to an isolate from chicken meat (within the same HeirC5 cgMLST cluster) than to the other serovar Hadar isolates we isolated (Supplementary Figure S2C). Serovar Typhimurium and Kentucky were both isolated from the same fecal sample (F26) and both isolates were most closely related to isolates from chicken (each was within the same HeirC5 cluster of a chicken isolate). Serovar Newport was identified four times, including two different Newport isolates from the same fecal sample (F7-5). Interestingly, while the closest whole genome match to these isolates was a single human isolate, they were also closely related to a number of serovar Newport isolates collected from surface waters in Georgia in 2011 (Supplementary Figure S3). For isolates belonging to serovars Give, Mississippi, Saintpaul, and Muenchen there were no other isolates in Enterobase that aligned closely, which limits assessment of potential sources for these isolates.

**Table 3:** Bird *Salmonella* isolates are related to isolates from a variety of sources.

Sample ID	Serovar	Farm Collected	Most closely related source type (genomic distance)	Isolate reference
JSBird1	Typhimurium	F5	Chicken (3)	SRR10883419
JSBird2	Kentucky	F5	Chicken (3)	SRR21413100
JSBird3	Hadar	F1	Turkey (2)	SRR3664900
JSBird4	Hadar	F2	Turkey (2)	SRR3664900
JSBird5	Hadar	F2	Turkey (2)	SRR3664900
JSBird6	Give	F8	River Water (24)	SRR2050944
JSBird7	Give	F18	River Water (24)	SRR2050944
JSBird8	Give	F18	River Water (23)	SRR2050944
JSBird9	Give	F18	River Water (23)	SRR2050944
JSBird10	Hadar	F9	Turkey (2)	SRR3664900
JSBird11	Hadar	F26	Chicken (5)	SRR1122614
JSBird12	Muenchen	F7	No Similarity	N/A
JSBird13	Newport	F7	Human (8)	SRR1646204
JSBird14	Saintpaul	F7	Human (29)	SRR6231044
JSBird15	Newport	F7	Human (7)	SRR1646204
JSBird16	Mississippi	F23	Human (46)	SRR9640338
JSBird17	Newport	F7	Human (5)	SRR16925338
JSBird18	Newport	F7	Human (7)	SRR1646204
JSBird19	Saintpaul	F7	Human (30)	SRR6231044

**Figure 3:**
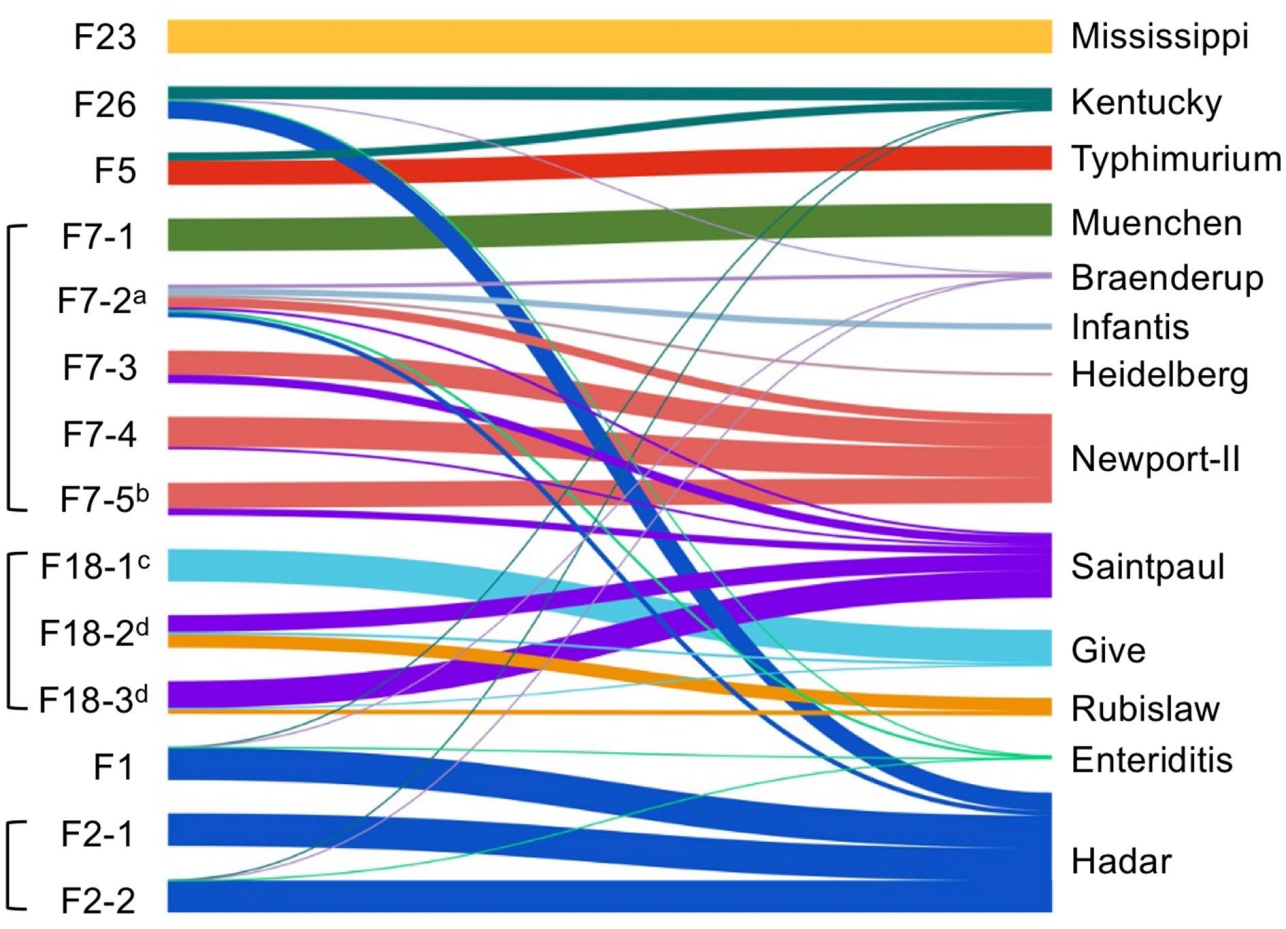
Multiserovar *Salmonella* populations exist in wild bird feces. A Sankey plot showing the sample (left nodes, indicated by the farm where the sample was collected) and the *Salmonella* serovar population within each sample. The colored bars represent different serovars (right nodes) and the thickness of the bars represent the relative abundance of each serovar within a population. Brackets around samples indicate that samples were collected from the same farm on the same day. For samples with a superscript alphabet, we were able to determine the bird species: ^a^chipping sparrow, ^b^house sparrow, ^c^cattle egret, and ^d^fish crow.

Deep serotyping by CRISPR-SeroSeq was performed on 14 samples. Two libraries failed to produce enough sequence reads, despite two attempts, and these both came from dry fecal samples. In total, 13 different serovars were identified (Figure 3). In these samples, 71% (10/14) had *Salmonella* populations consisting of multiple serovars, with an average of 2.6 serovars per sample (range, 1–7 serovars per sample). Serovars included Saintpaul (*n* = 6), Hadar (*n* = 5), Newport (*n* = 4), Kentucky (*n* = 4), Enteritidis (*n* = 4), Braenderup (*n* = 4), Give (*n* = 3), Rubislaw (*n* = 2), Heidelberg (*n* = 1), Infantis (*n* = 1), Muenchen (*n* = 1), Typhimurium (*n* = 1), and Mississippi (*n* = 1). Importantly, serovars Enteritidis, Infantis, and Braenderup, which were in the top 10 serovars found to cause human illness between 2019–2021 (Centers for Disease Control and Prevention, 2022), were always outnumbered by other serovars when they were present (outnumbered serovars have thinner connecting lines in Figure 3), and unsurprisingly, we did not isolate these by culture. In congruence with our whole genome sequence analyses, samples collected from the same sites on the same days often contained similar *Salmonella* populations. For example, two of the three fecal samples collected from farm 18 (F18-2,3) had nearly identical *Salmonella* profiles (serovars Saintpaul, Rubislaw, and Give) with respect to the serovars that were present and their relative frequency within each sample.

Point counts were performed at each farm visit and identified 1,123 individuals. This included 51 species, with the most prevalent being the northern cardinal (*Cardinalis cardinalis*) (*n* = 48 visits where species was observed), the northern mockingbird (*Mimus polyglottos*) (*n* = 46), and the barn swallow (*Hirundo rustica*) (*n* = 45) (Table 4). A total of 31 species were observed in-field, with the most common being the song sparrow (*Melospiza melodia*) (*n* = 19), eastern phoebe (*Sayornis phoebe*) (*n* = 14), northern cardinal (*n* = 13), chipping sparrow (*Spizella passerine*) (*n* = 13), and mourning dove (*Zenaida macroura*) (*n* = 11). Of these, *Salmonella* was detected in four species, including three times from chipping sparrows. Off-field species included the barn swallow (*n* = 39), northern mockingbird (*n* = 38), and the Carolina wren (*Thryothorus ludovicianus*) (*n* = 38). Notably, some species were not often identified, but when present, were found in large numbers. For example, the rock pigeon (*Columba livia*) was only observed during four visits, but 147 individuals were recorded (Supplementary Figure S4). Rock pigeons were not in the top ten most frequently observed bird species across this study; however, they were the first and second highest in terms of total individuals off-field and in-field, respectively. Similarly, the common grackle (*Quiscalus quiscula*) was also observed off-field during four visits, but 83 individuals were recorded. Molecular species identification was done *via* PCR and sequencing of the COI gene was completed on 161 (20.8%) samples. This identified 24 species with the most common being the eastern bluebird (*Sialia sialis*) (*n* = 36) and the northern mockingbird (*n* = 19) (Table 4). The individuals that were culture-positive for *Salmonella* were a chipping sparrow, an eastern bluebird, a cattle egret (*Bubulcus ibis*), a house sparrow (*Passer domesticus*), and two fish crows (*Corvus ossifragus*). Because we only identified the bird species in 13 *Salmonella*-positive fecal samples, conclusions based on the *Salmonella* status of specific bird species are limited.

**Table 4:** Bird species identified by bird counts and by molecular analysis.

Species	In field		Off field		Total species observations	Molecular observations (COI)	*Salmonella* culture	*Salmonella* PCR	Total *Salmonella* positive
	Number of species observations	Total number of individuals	Number of species observations	Total number of individuals					
Song sparrow (*Melospiza melodia*)	19	33	6	8	25	0	0	0	0
Eastern phoebe (*Sayornis phoebe*)	14	16	21	23	35	5	0	1	1
Northern cardinal (*Cardinalis cardinalis*)	13	25	35	41	48	14	0	1	1
Chipping sparrow (*Spizella passerina*)	13	16	22	32	35	9	1	2	3
Mourning dove (*Zenaida macroura*)	11	67	26	44	37	9	0	1	1
Northern mockingbird (*Mimus polyglottos*)	8	13	38	47	46	19	0	0	0
Eastern bluebird (*Sialia sialis*)	7	11	15	25	22	36	1	0	1
Barn swallow (*Hirundo rustica*)	6	18	39	59	45	0	0	0	0
Killdeer (*Charadrius vociferus*)	6	6	2	4	8	0	0	0	0
Red-winged blackbird (*Agelaius phoeniceus*)	5	5	4	7	9	7	0	0	0
House finch (*Haemorhous mexicanus*)	4	8	19	56	23	11	0	1	1
Eastern meadowlark (*Sturnella magna*)	4	4	2	3	6	0	0	0	0
Indigo bunting (*Passerina cyanea*)	4	4	0	0	4	10	0	1	1
American Crow *(Corvus brachyrhynchos)*	3	8	16	29	19	12	0	0	0
European collared dove (*Streptopelia decaocto*)	3	4	2	2	5	0	0	0	0
American goldfinch (*Spinus tristis*)	3	3	4	4	7	0	0	0	0
Rock pigeon (*Columba livia*)	2	45	2	102	4	0	0	0	0
Cattle egret (*Bubulcus ibis*)	2	13	2	5	4	1	1	0	1
Carolina wren (*Thryothorus ludovicianus*)	2	3	38	46	40	0	0	0	0
Field sparrow (*Spizella pusilla*)	2	3	6	6	8	1	0	0	0
Eastern kingbird (*Tyrannus tyrannus*)	2	3	1	1	3	2	0	0	0
Blue jay (*Cyanocitta cristata*)	2	2	20	25	22	0	0	0	0
Tufted titmouse (*Baeolophus bicolor*)	2	2	9	9	11	0	0	0	0
Ruby-throated hummingbird (*Archilochus colubris*)	2	2	0	0	2	0	0	0	0
Eurasian collared dove (*Streptopelia decaocto*)	1	6	0	1	1	0	0	0	0
European starling (*Sturnus vulgaris*)	1	3	4	16	5	3	0	0	0
Common ground dove (*Columbina passerina*)	1	2	1	2	2	0	0	0	0
White-eyed vireo (*Vireo griseus*)	1	1	10	10	11	0	0	0	0
Blue grosbeak (*Passerina caerulea*)	1	1	7	7	8	7	0	0	0
Brown thrasher (*Toxostoma rufum*)	1	1	5	5	6	0	0	0	0
Downy woodpecker (*Picoides pubescens*)	1	1	5	5	6	0	0	0	0
Eastern towhee (*Pipilo erythrophthalmus*)	0	0	11	13	11	0	0	0	0
Red-bellied woodpecker (*Melanerpes carolinus*)	0	0	7	9	7	0	0	0	0
Carolina chickadee (*Poecile carolinensis*)	0	0	5	12	5	0	0	0	0
Chimney swift (*Chaetura pelagica*)	0	0	5	11	5	1	0	0	0
Common grackle (*Quiscalus quiscula*)	0	0	4	83	4	0	0	0	0
Red-shouldered hawk (*Buteo lineatus*)	0	0	4	6	4	0	0	0	0
Pine warbler (*Setophaga pinus*)	0	0	3	3	3	0	0	0	0
Black vulture (*Coragyps atratus*)	0	0	2	9	2	0	0	0	0
American robin (*Turdus migratorius*)	0	0	2	6	2	0	0	0	0
American kestrel (*Falco sparverius*)	0	0	2	2	2	0	0	0	0
Brown-headed nuthatch (*Sitta pusilla*)	0	0	2	2	2	0	0	0	0
Painted bunting (*Passerina ciris*)	0	0	2	2	2	1	0	0	0
Yellow-throated vireo (*Vireo flavifrons*)	0	0	2	2	2	0	0	0	0
Northern rough-winged swallow (*Stelgidopteryx serripennis*)	0	0	1	4	1	0	0	0	0
House sparrow (*Passer domesticus*)	0	0	1	1	1	5	1	0	1
Northern parula (*Setophaga americana*)	0	0	1	1	1	0	0	0	0
Pileated woodpecker (*Dryocopus pileatus*)	0	0	1	1	1	0	0	0	0
Red-tailed hawk (*Buteo jamaicensis*)	0	0	1	1	1	0	0	0	0
Tree swallow (*Tachycineta bicolor*)	0	0	1	1	1	0	0	0	0
White-breasted nuthatch (*Sitta carolinensis*)	0	0	1	1	1	0	0	0	0
Blue-gray gnatcatcher (*Polioptila caerulea*)	0	0	0	0	0	1	0	0	0
Brown-headed cowbird (*Molothrus ater*)	0	0	0	0	0	2	0	0	0
Eastern wood pewee (*Contopus virens*)	0	0	0	0	0	1	0	0	0
Fish crow (*Corvus ossifragus*)	0	0	0	0	0	2	2	0	2
Great crested flycatcher (*Myiarchus crinitus*)	0	0	0	0	0	1	0	0	0
Summer tanager (*Piranga rubra*)	0	0	0	0	0	1	0	0	0
Total	146	329	419	794	565	161	6	7	13

## 4. Discussion

This study investigated the impact of wild birds on food safety by surveying *Salmonella* in wild bird feces deposited on foliage on produce farms over a two-year period in the Southeast. Our study demonstrated that the overall prevalence of culturable *Salmonella* in the Southeast was 2.1%, but this differed greatly between 2021 (6.6%) and 2022 (0.2%). Studies have been completed in other regions include the west coast where *Salmonella* prevalence ranged from 0.5% in cultured fecal samples (Gorski et al., 2011; Franklin and VerCauteren, 2016; Navarro-Gonzalez et al., 2020; Smith et al., 2020) to 2.5% in cultured bird gastrointestinal tracts (Kirk et al., 2002), and the Southwest where one study found a 1.9% prevalence in bird feces (Rivadeneira et al., 2016). Other studies outside of the US have included Europe (Palmgren et al., 2006; Lawson et al., 2011), South America (Cardoso et al., 2021), and the Middle East (Cohen et al., 2021). The overall prevalence identified in the current study aligns with this body of literature. Unlike most studies that sampled fresh feces (i.e., collected directly from a bird), this study offered us the opportunity to evaluate whether *Salmonella* is likely to be recovered from defecated material on foliage. *Salmonella* was isolated by culture and also detected by PCR three times more frequently in moist feces (presumably deposited within a few hours of collection) compared to dry feces. This suggests that *Salmonella* survival in feces is dynamic and the population reduces as the feces dry. While prior work has shown *Salmonella* can survive in feces up to 291 days (Topalcengiz et al., 2020) and can have improved survival in low moisture environments (Oni et al., 2015), these studies were performed in controlled laboratory experiments and do not necessarily reflect conditions in a produce field. The fecal samples we collected had a much larger surface area to volume ratio, therefore are likely to dry out faster than homogenized laboratory samples.

Our statistical models suggest that precipitation the day before sampling positively influences the moisture of wild bird fecal samples, which is expected. Comparison of precipitation during sampling months in both years supports this relationship, with lower precipitation in 2022 than in 2021 likely accounting for decreased moisture and therefore a reduced *Salmonella* recovery. One model included a negative correlation between increased wind and moist feces, which is also expected as increased wind would dry the feces more rapidly. Alternatively, humidity showed a negative influence on fecal moisture in three different models. This seems counterintuitive; however one study of *Salmonella* survival in a controlled environment also saw a negative association between humidity and pathogen recovery from turkey feces (Oni et al., 2015).

Most studies of *Salmonella* in wild birds have involved capturing birds and collecting fresh feces or swabbing the cloaca (Gorski et al., 2011; Hernandez et al., 2016; Navarro-Gonzalez et al., 2020; Murray et al., 2021) while others have applied molecular techniques (i.e., PCR) to identify *Salmonella* in bird feces (Rivadeneira et al., 2016; Smith et al., 2020; Zhao et al., 2020; Olimpi et al., 2022). PCR is a very sensitive method for pathogen detection, and we detected nearly five times as many *Salmonella-*positive fecal samples when we used PCR compared to culture. *Salmonella* has been shown to be detectable by PCR up to 10 days after inoculation; however, significant reduction occurs after four days (Lopez-Velasco et al., 2015). There are three possibilities that could explain the discrepancy between the culture results and the PCR results: (i) PCR can detect dead *Salmonella*; (ii) PCR can detect viable but non-culturable (VBNC) *Salmonella*; and (iii) because PCR is more sensitive than culture, it is possible that where the amount of *Salmonella* in the feces was very low, we were not able to recover it from culture but could detect it by PCR. We note that the background microflora was not particularly high in the selective enrichment broths nor on the XLT-4 plates, so we do not suspect that this contributed to not being able to detect *Salmonella* via our culture methods. We did attempt to serotype the PCR-detected *Salmonella* using the ISR method (Guard et al., 2022) to determine whether there were any serovar associations with PCR versus culture, but we were unsuccessful. The *Salmonella* detected by culture may have been present in higher loads, which allowed us to isolate it more easily, although we did not quantify *Salmonella*. Whether the PCR-only positive samples represent VBNC cells and pose a food safety risk should be a focus of future studies, especially as PCR-based diagnostic assays are more commonly being used to screen food products.

In addition to completing a surveillance study, this work also assessed the need for and efficacy of no-harvest buffer zones around feces in a production environment (Hamilton et al., 2015). While produce directly contacting feces cannot be harvested, the Produce Safety Rule does not require the establishment of no-harvest buffer zones, nor does it recommend suggested distances surrounding contaminated produce to exclude from harvest. Depending on the recommended buffer zone radius and the impacted commodity type, buffer recommendations could have a substantial economic impact on growers and could be excluding produce that is safe for consumption. *Salmonella* was not isolated from additional plant samples below foliage with fecal contamination nor from neighboring plants downwind. However, depending on the weather or other climate factors, the rate at which feces dries on the plant surface may vary; this may be important to consider since our data shows that culturable *Salmonella* is primarily present in moist feces. The low incidence of *Salmonella* in bird feces and the lack of evidence supporting spread to adjacent plants in this study may be useful data for growers as they establish procedures for managing bird feces before and during harvest.

Alongside determining *Salmonella* prevalence in bird fecal samples, a deeper analysis was conducted into individual *Salmonella* isolates and serovar populations. Previous work has shown a high level of diversity within bird feces, including identifying as many as three serovars of *Salmonella* from a single sample (Antilles et al., 2021). Our culture-based analysis supported this high diversity by identifying eight serovars among 19 isolates. High-resolution analysis by deep serotyping revealed even great serovar diversity, by detecting 13 serovars across 14 samples. Further, we showed that 62.5% of culture positive samples contain multiple serovars, which included one fecal sample that contained seven different serovars (F7-2). Six serovars identified here (serovars Enteriditis, Newport, Typhimurium, Infantis, Saintpaul, and Muenchen) were determined by the Centers for Disease Control (CDC) to be among the top 10 serovars associated with human illness between 2019–2021 (Centers for Disease Control and Prevention, 2022). Additionally, serovars Hadar, Heidelberg, and Braenderup have all been linked to human outbreaks in produce or animal products in the past ten years (CDC, 2023) and were also found in our samples. Importantly, serovars Enteritidis and Braenderup were each found in four different fecal samples and in each of these, they were significantly outnumbered by other serovars that are not known to be associated with human foodborne illness. For example, in one sample (F2-2), serovar Hadar constituted 95.9% of the total *Salmonella* and serovar Enteritidis was only 0.1%. As our results demonstrated, using traditional culture-based *Salmonella* isolation, serovars Enteritidis and Braenderup were never detected, indicating that these important serovars were overlooked. From the five serovar Hadar isolates identified, four were closely related (within the same hierCC 2 cluster on Enterobase), to isolates from commercial turkeys (Supplementary Figure S1B). It should be noted that there is no commercial turkey production within at least 200 miles of the location of these farms and the turkey isolates were from 2012–2016. The fifth serovar Hadar isolate was related (within the same hierCC 5 cluster) to a chicken isolate, though that isolate was collected in 2015 from Oregon. While chicken production in the southeast is well established, further research is needed to determine whether and how wild birds acquire *Salmonella* from commercial poultry operations (e.g., from foraging on poultry farms, or from encountering contaminated poultry manure on produce farms). Interestingly, the four serovar Newport isolates most closely matched to human isolates; however, they were also closely related to isolates collected from fresh water sources in Georgia (Supplementary Figure S2). Two different Newport isolates came from the same fecal sample (F7-5), where one was identified from each selective enrichment broth, indicating there is also strain diversity within single fecal samples. Deep serotyping showed that samples collected from the same farm often had similar *Salmonella* serovar populations in addition to closely related isolates, suggesting that similar sources of *Salmonella* may occur in the environment that contribute to contamination in wild birds, or that a single bird was defecating multiple times in the same field. Alternatively, for birds that flock together (e.g., crows), this similarity may reflect transmission within a flock, for example at common feeding or watering locations. Overall, our findings indicate that wild birds have the potential to obtain and transmit *Salmonella* from a wide range of sources over large geographic areas.

Bird species were identified in this study using both physical and molecular methods. Other studies have used a more direct collection approach where birds are caught using nets or traps followed by the collection of feces or swabbing the cloaca (Gruszynski et al., 2014; Fuentes-Castillo et al., 2019; Navarro-Gonzalez et al., 2020). In these instances, bird species can be identified quite easily, and the sample is fresher. Alternatively, the collection method used in this study resulted in lower molecular characterization of bird species (20.8% identified). However, it was non-invasive and provided an opportunity to investigate bird species actively defecating on the field, not just those primarily foraging in adjacent habitats. Data collected in this study identified 51 species of bird from point counts and 24 species from COI, for a total of 57 species. Molecular detection from bird feces allowed for the identification of six additional species, including the fish crow, which was identified in two *Salmonella* culture positive samples but not identified during point counts. This demonstrates the importance of the two complementary methods for bird identification.

We categorized birds from our point counts as in-field or off-field. The off-field category included species that are often associated with agricultural structures (e.g., barns, packing houses, fences) or other structures (e.g., powerlines adjacent to the farm), such as the barn swallow (*n* = 39 species observations off-field), house finch (*Haemorhous mexicanus*) (*n* = 19), European starling (*Sturnus vulgaris*) (*n* = 4), and rock pigeon (*n* = 2). Of these, only a single house finch fecal sample tested positive for *Salmonella.* Although rock pigeons were observed twice off-field, the total number of individuals was 102, suggesting that flock size may also be relevant with respect to understanding the risk posed by different species. The off-field category also included birds found away from the farm premises (e.g., in tree line, neighboring pasture) and included the Carolina wren (*n* = 26), woodpecker (*n* = 12), white-eyed vireo (*Vireo griseus*) (*n* = 8), and eastern towhee (*Pipilo erythrophthalmus*) (*n* = 3). This latter group poses the lowest risk of pathogen transmission because they are infrequently observed interacting with produce. Although we were not able to identify the bird species for the majority of *Salmonella* positive fecal samples, of the ones we were able to identify, none belonged to this category. Conversely, birds on agricultural structures and in-field pose a higher food safety risk because of their interactions with farm livestock and produce, so deterrents targeting these species would be more effective. Three quarters (10/13) of *Salmonella-*positive fecal samples were from birds that were also observed as in-field during point counts. For the three that were not observed, one was a cattle egret and the other two were fish crows. Interestingly, fish crows are associated with water and both had *Salmonella* serovars Give and Rubislaw (F18-2, -3), which are associated with surface water (Haley et al., 2009; Gorski et al., 2011; McEgan et al., 2014; Maurer et al., 2015; Callahan et al., 2019; Deaven et al., 2021).

Mitigating risks associated with wild birds in produce fields remains a complicated issue that will require a One Health approach to fully understand how the interaction of animals (including wildlife, such as birds, and food animals), the environment, and human activity contribute to *Salmonella* ecology. In this work, we found a low prevalence of *Salmonella*, however; serovars associated with human illness were often identified when *Salmonella* was present. Moreover, the prevalence increased from 2% to over 9% when molecular detection was included, suggesting that different methods of detection can influence the establishment of risk due to this environmental source of *Salmonella.* The complexity of this problem is highlighted by our whole genome analysis showing that *Salmonella* isolates recovered in this study were related within 10 pairwise allelic differences (PADs) to isolates from a range of sources including humans, animal agriculture, and the environment, as well as some without any links to these sources. The freshness of the wild bid feces was shown to impact viability of *Salmonella*; however, more work will need to be completed to show how risk of feces changes with time and if certain serovars are better adapted to this environment. While factors affecting the prevalence of *Salmonella* within wild birds and *Salmonella* survival within feces are not fully understood, the findings presented here contribute to our understanding of these complex food safety systems.

## Data availability statement

The datasets presented in this study can be found in online repositories. The names of the repository/repositories and accession number(s) can be found in the article.

## Author contributions

JCS: Data curation, Formal analysis, Investigation, Writing – original draft, Writing – review & editing, Visualization. SV: Data curation, Formal analysis, Investigation, Writing – review & editing. KR: Resources, Writing – review & editing. ZS: Resources, Writing – review & editing. JD: Resources, Writing – review & editing. JS: Resources, Writing – review & editing. LD: Conceptualization, Funding acquisition, Investigation, Methodology, Project administration, Resources, Supervision, Writing – review & editing. WS: Conceptualization, Funding acquisition, Investigation, Methodology, Project administration, Supervision, Writing – review & editing. NS: Conceptualization, Data curation, Formal analysis, Funding acquisition, Investigation, Methodology, Project administration, Supervision, Writing – original draft, Writing – review & editing.
