# Supplementary material for: Prevalence and molecular characterization of Salmonella isolated from wild birds in fresh produce environments
Source: Front Microbiol. 2023 Nov 7;14:1272916. doi: 10.3389/fmicb.2023.1272916 (PMC10662084; doi:10.3389/fmicb.2023.1272916)
Supplement: Supplementary file 1 [file Table_1.pdf]

Supplemental Table 1. ΔAICc and Akaike weight (ω) for detection of *Salmonella* and weather effect models.

Model	β Value (SE, p)			ΔAICc	ω
	Precipitation	Humidity	Wind		
Humidity		-0.2898 (0.179, 0.105)		0	0.379
Precipitation + Humidity	0.2348 (0.190, 0.215)	-0.3375 (0.183, 0.065)		0.45	0.289
Precipitation + Humidity + Wind	0.2788 (0.197, 0.158)	-0.2833 (0.190, 0.129)	-0.2006 (0.208, 0.335)	1.54	0.169
Precipitation	0.165 (0.189, 0.381)			1.88	0.139
