# Supplementary material for: Prevalence and molecular characterization of Salmonella isolated from wild birds in fresh produce environments
Source: Front Microbiol. 2023 Nov 7;14:1272916. doi: 10.3389/fmicb.2023.1272916 (PMC10662084; doi:10.3389/fmicb.2023.1272916)
Supplement: Supplementary file 2 [file Table_2.pdf]

Supplemental Table 2. Genome assembly statistics

	Genome Size (Mb)	Number of contigs	N50 (Kb) Value
JSBird1	4.95	132	103.59
JSBird2	4.99	193	66.87
JSBird3	4.74	129	114.65
JSBird4	4.74	122	115.51
JSBird5	4.75	40	735.89
JSBird6	4.77	101	140.49
JSBird7	4.77	102	159.79
JSBird8	4.77	137	98.89
JSBird9	4.77	122	127.85
JSBird10	4.74	121	94.07
JSBird11	4.67	37	613.89
JSBird12	5.03	129	101.89
JSBird13	4.84	184	86.61
JSBird14	4.91	177	71.96
JSBird15	4.84	149	102.96
JSBird16	4.61	268	36.06
JSBird19	4.80	55	405.57
JSBird20	4.85	57	430.46
JSBird21	4.92	59	417.46
