# Supplementary material for: Prevalence and molecular characterization of Salmonella isolated from wild birds in fresh produce environments
Source: Front Microbiol. 2023 Nov 7;14:1272916. doi: 10.3389/fmicb.2023.1272916 (PMC10662084; doi:10.3389/fmicb.2023.1272916)
Supplement: Supplementary file 3 [file Image_1.pdf]

| North Georgia | May | June | July | August | September | October |
|---|---|---|---|---|---|---|
| Temperature (°F) | 3.88 | 4.59 | 2.05 | -0.83 | 0.56 | -1.71 |
| Precipitation (in) | -1.41 | -3.05 | 0.12 | -2.69 | -0.96 | -5.49 |
| Humidity (%) | 4.61 | -8.84 | -1.18 | 2.95 | -8.58 | -13.71 |
| Wind (mph) | -0.09 | 0.19 | 0.45 | -0.55 | 1.37 | 0.93 |

*Sampling in 2021 took place between June 13th-October 14th
*Sampling in 2022 took place between May 31st-August 24th

| South Georgia/Florida | May | June | July | August | September | October |
|---|---|---|---|---|---|---|
| Temperature (°F) | 2.12 | 4.32 | 2.38 | 1.00 | 0.11 | -0.48 |
| Precipitation (in) | 4.82 | -2.94 | -0.89 | -3.08 | -2.34 | -1.10 |
| Humidity (%) | 9.48 | -3.68 | -2.43 | -2.79 | -6.06 | -9.24 |
| Wind (mph) | -0.87 | -0.19 | 0.11 | -1.27 | 1.86 | 0.59 |

*Sampling in 2021 took place between May 26th-September 18th
*Sampling in 2022 took place between May 23rd-June 29th

| Tennessee | May | June | July | August | September | October |
|---|---|---|---|---|---|---|
| Temperature (°F) | 4.13 | 3.23 | 0.97 | -1.06 | 0.17 | -2.87 |
| Precipitation (in) | 0.04 | -0.03 | 0.20 | -0.24 | 0.03 | -0.08 |
| Humidity (%) | 2.91 | -8.19 | 4.02 | 0.16 | -7.25 | -19.50 |
| Wind (mph) | 0.64 | 0.47 | 0.63 | 0.23 | 1.12 | 0.81 |

*Sampling in 2021 took place between June 23rd-October 1st
*Sampling in 2022 took place between June 22nd-August 30th

| South Carolina | May | June | July | August | September | October |
|---|---|---|---|---|---|---|
| Temperature (°F) | 2.69 | 5.00 | 2.55 | -1.05 | -1.57 | -4.48 |
| Precipitation (in) | 0.30 | -1.50 | 0.94 | -0.28 | -1.10 | -1.42 |
| Humidity (%) | 4.40 | -7.37 | 2.41 | 0.04 | -2.27 | -6.38 |
| Wind (mph) | 0.62 | -0.83 | 0.86 | 0.16 | 1.32 | -0.01 |

*Sampling in 2021 took place between August 3rd-October 21st
*Sampling in 2022 took place between June 9th-August 10th

**Supplemental Figure 1. Comparison of weather conditions for sampling regions.** Temperature, precipitation, humidity, and wind were compared between 2021 and 2022 collection seasons. Averages for each variable (except precipitation where cumulative rainfall was calculated) are shown in under the respective month for each region. Negative numbers (red boxes) indicate a lower value in 2022 and positive numbers (blue boxes) indicate a higher value in 2022. Dates of the first and last sampling in each region for both years are listed to the right of the weather values.
