# Supplementary material for: Prevalence and molecular characterization of Salmonella isolated from wild birds in fresh produce environments
Source: Front Microbiol. 2023 Nov 7;14:1272916. doi: 10.3389/fmicb.2023.1272916 (PMC10662084; doi:10.3389/fmicb.2023.1272916)
Supplement: Supplementary file 4 [file Image_2.pdf]

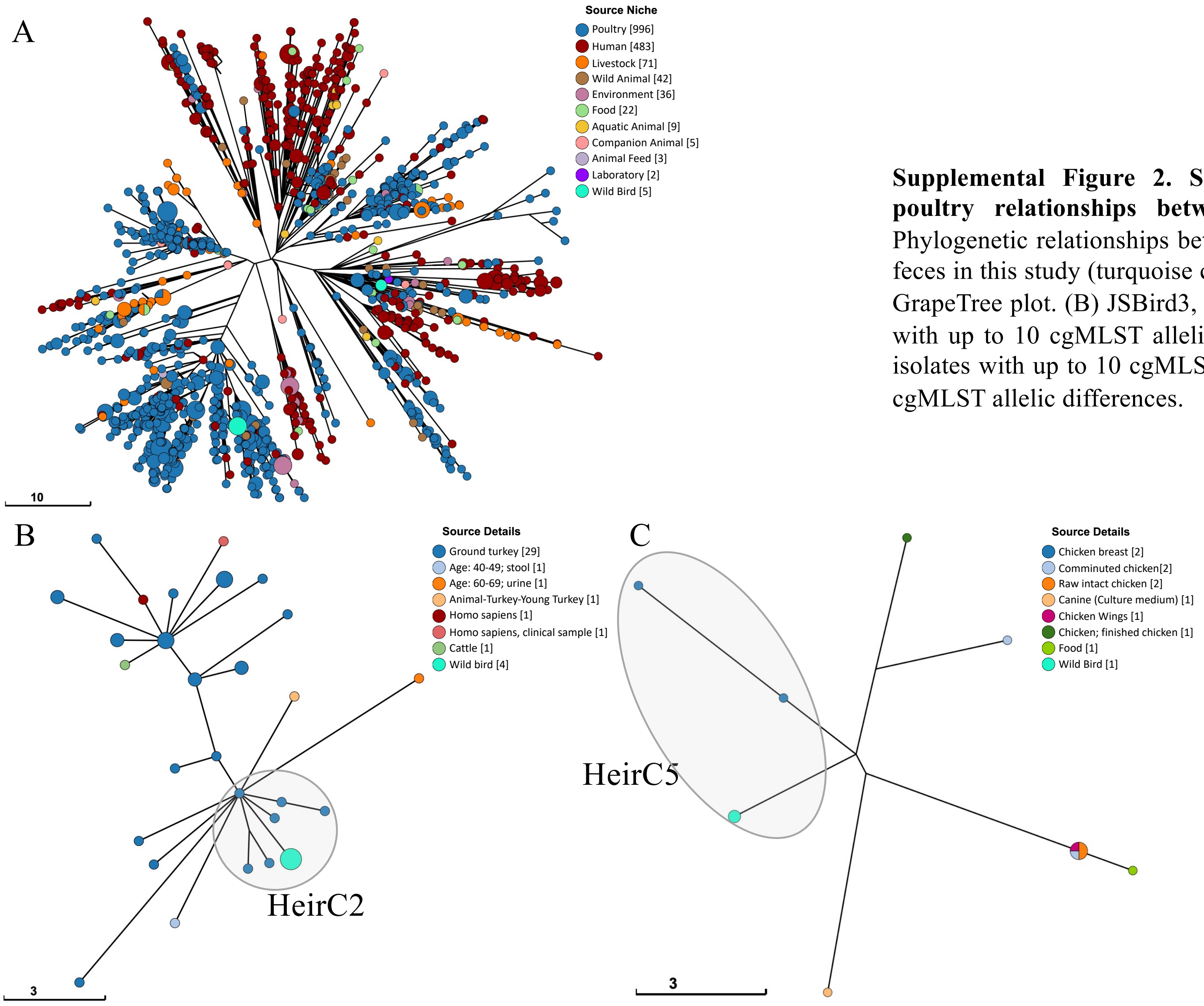

Supplemental Figure 2. Serovar Hadar isolates include variable poultry relationships between turkey and chicken isolates. (A) Phylogenetic relationships between serovar Hadar isolates from wild bird feces in this study (turquoise circles) to isolates on Enterobase shown on a GrapeTree plot. (B) JSBird3, 4, 5, and 10 isolates and Enterobase isolates with up to 10 cgMLST allelic differences. (C) JSBird11 and Enterobase isolates with up to 10 cgMLST allelic differences. (Scale bar) Number of cgMLST allelic differences.
