# Supplementary material for: Prevalence and molecular characterization of Salmonella isolated from wild birds in fresh produce environments
Source: Front Microbiol. 2023 Nov 7;14:1272916. doi: 10.3389/fmicb.2023.1272916 (PMC10662084; doi:10.3389/fmicb.2023.1272916)
Supplement: Supplementary file 5 [file Image_3.pdf]

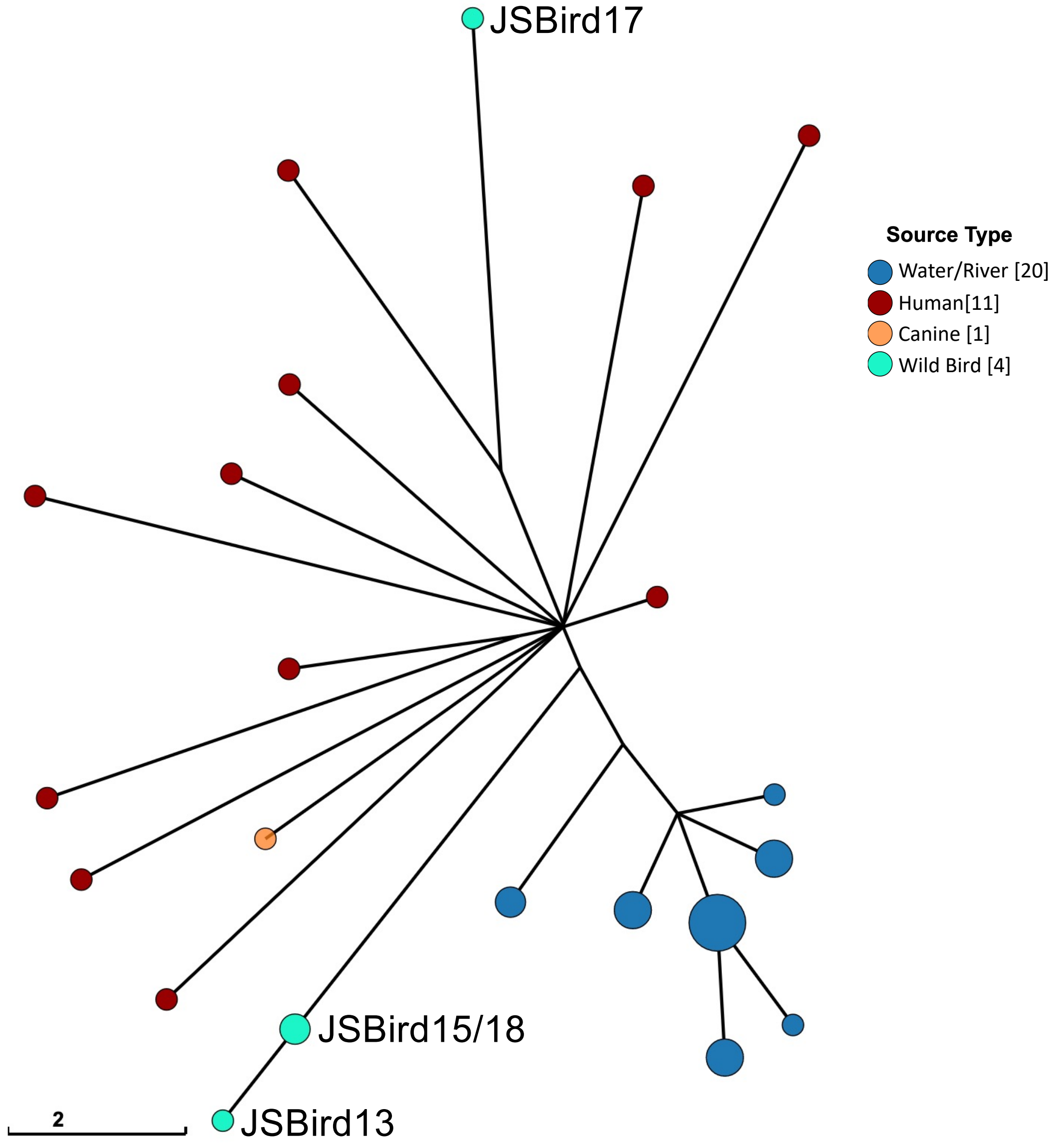

Supplemental Figure 3. Serovar Newport isolates are similar to human and environmental isolates. Serovar Newport isolates from wild bird feces (turquise circles) with phylogenetic relationships to isolates within Enterobase shown on a GrapeTree plot. (Scale bar) Number of cgMLST allelic differences.
