# Supplementary material for: Prevalence and molecular characterization of Salmonella isolated from wild birds in fresh produce environments
Source: Front Microbiol. 2023 Nov 7;14:1272916. doi: 10.3389/fmicb.2023.1272916 (PMC10662084; doi:10.3389/fmicb.2023.1272916)
Supplement: Supplementary file 6 [file Image_4.pdf]

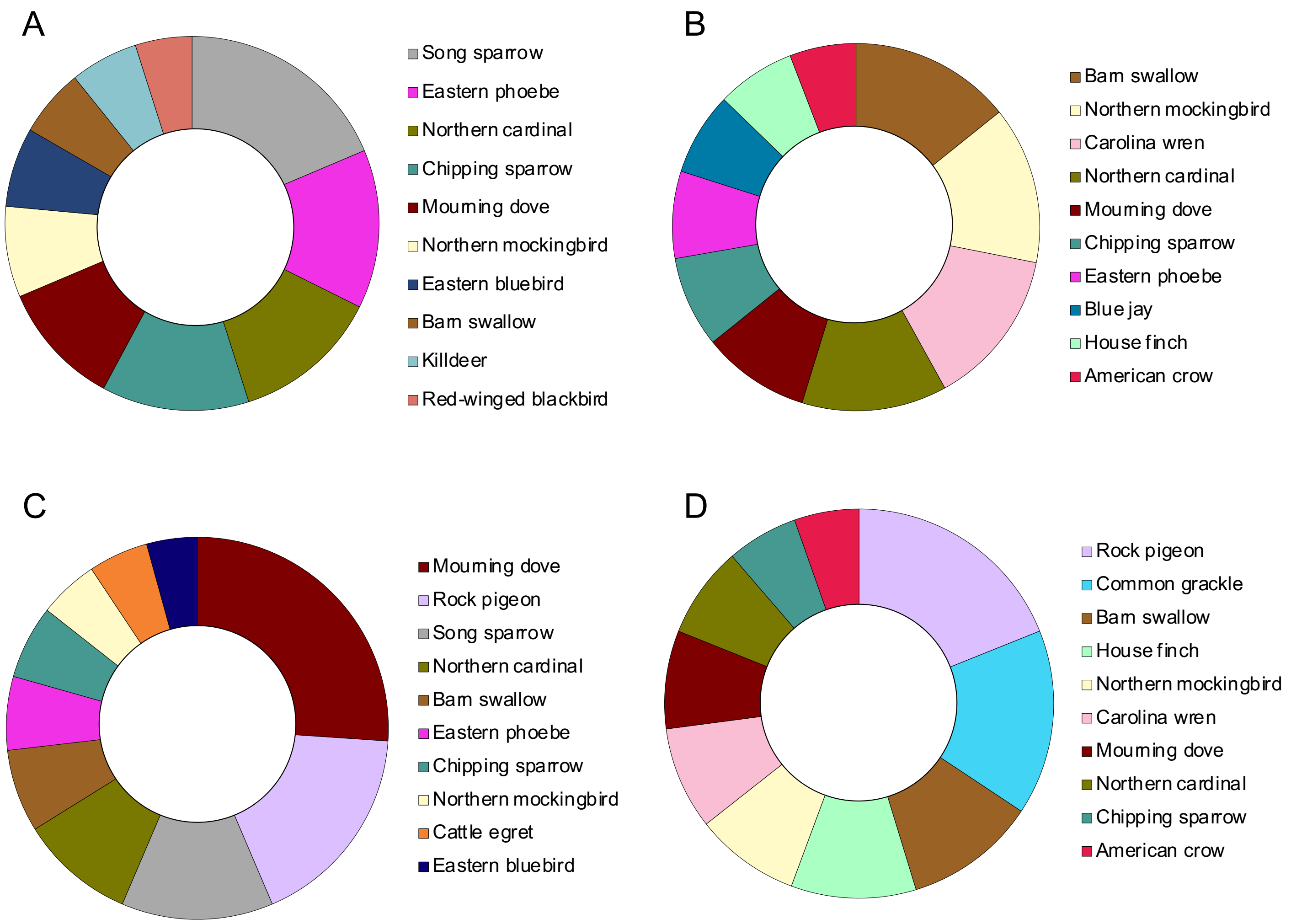

Supplemental Figure 4. Wild bird point counts. Top 10 species for the following categories: (A) Visits with species observation in-field, (B) visits with species observation off-field, (C) in-field individual observations, and (D) off-field individual observations.
